# Evaluation of Foot-and-Mouth Disease (FMD) Virus Asia1 Genotype-V as an FMD Vaccine Candidate: Study on Vaccine Antigen Production Yield and Inactivation Kinetics

**DOI:** 10.3390/vaccines12020185

**Published:** 2024-02-12

**Authors:** Jae Young Kim, Sun Young Park, Sang Hyun Park, Gyeongmin Lee, Jong-Sook Jin, Dohyun Kim, Jong-Hyeon Park, Seong-Yun Jeong, Young-Joon Ko

**Affiliations:** 1 Center for FMD Vaccine Research, Animal and Plant Quarantine Agency, Gimcheon-si 177, Republic of Korea; ivorikim@korea.kr (J.Y.K.); sun3730@korea.kr (S.Y.P.); shpark0205@korea.kr (S.H.P.); lgm6004@korea.kr (G.L.); in75724@korea.kr (J.-S.J.); doh936@korea.kr (D.K.); parkjhvet@korea.kr (J.-H.P.); 2 Department of Biomedical Science, Graduate School, Catholic University of Daegu, Daegu 38430, Republic of Korea; jsymicro@cu.ac.kr

**Keywords:** foot-and-mouth disease, Asia1/MOG/05, vaccine, antigen, inactivation

## Abstract

South Korea has experienced outbreaks of foot-and-mouth disease (FMD) of serotypes O and A, leading to nationwide vaccination with a bivalent vaccine. Since the FMD virus (FMDV) Asia1 group-V genotype occurred in North Korea in 2007, an Asia1/MOG/05 vaccine strain belonging to the Asia1 group-V genotype was developed using a genetic recombination method (Asia1/MOG/05-R). This study aimed to evaluate the antigen productivity and viral inactivation kinetics of Asia1/MOG/05-R to assess its commercial viability. The antigen yield of Asia1/MOG/05-R produced in flasks and bioreactors was approximately 4.0 μg/mL. Binary ethylenimine (BEI) inactivation kinetics of Asia1/MOG/05-R showed that 2 mM and 1.0 mM BEI treatment at 26 °C and 37 °C, respectively, resulted in a virus titer <10^−7^ TCID_50_/mL within 24 h, meeting the inactivation kinetics criteria. During incubation at 26 °C and 37 °C, 10% antigen loss occurred, but not due to BEI treatment. When pigs were inoculated twice with the Asia1/MOG/05-R antigen, the virus neutralization titer increased to approximately 1:1000; therefore, it can sufficiently protect against Asia1/MOG/05-R and Asia1 Shamir viruses. The Asia1/MOG/05-R will be useful as a vaccine strain for domestic antigen banks.

## 1. Introduction

Foot-and-mouth disease (FMD) is a highly contagious disease affecting cloven-hoofed animals. Its substantial economic impact has increased the concerns of governments worldwide [1]. FMD virus (FMDV), an *Aphthovirus* genus of the *Picornaviridae* family, is the causative agent of FMD [2]. The nucleic acid of FMDV is a single-stranded, positive-sense RNA genome. The genes corresponding to the capsid are translated into a polyprotein, which is cleaved into structural and nonstructural proteins by proteases [1,3,4]. Of the seven FMD serotypes (O, A, Asia1, C, and SAT 1–3), types O, A, and Asia1 have been most commonly identified in Asian countries. Type Asia1 is grouped into nine genetic groups (G-I to G-IX) based on nucleotide variations in the VP1 sequence [5].

In South Korea, FMD broke out in February 2000, after the last FMD outbreak in 1934. Due to the overseas antigen bank established in advance, FMD was eliminated early through an emergency vaccination policy. However, two years later, FMD occurred again, but this time, it was successfully controlled through a culling policy. There was no FMD outbreak for a while, but in January 2010, FMD type A occurred, followed by type O in April, and then a massive scale outbreak of FMD type O occurred from November to April of the following year, causing about 3 trillion won in economic damage. Since then, FMD vaccines have been administered nationwide to cattle, pigs, goats etc. FMD occurred every year from 2014 to 2019 and then again in 2023. As FMD outbreaks have been occurring intermittently, the vaccination policy will need to be maintained to protect the domestic livestock industry. 

The serotypes of FMD that have occurred in South Korea so far are serotypes O and A, so a vaccine containing a mixture of the two serotypes is being used. Vaccines for the remaining five serotypes (Asia1, C, and SAT 1, SAT 2, SAT 3), which have never occurred in South Korea, are stockpiled in the form of an antigen bank under a contract with a foreign FMD vaccine manufacturing facility, and in the event of a domestic outbreak, the finished product will be delivered to South Korea within six working days according to the terms of the contract. 

Meanwhile, the current FMD vaccines are entirely imported from abroad. However, if an FMD vaccine manufacturing facility is built in South Korea in the next 2–3 years, localization of the FMD vaccine will be achieved, so for this purpose, our institute has been developing various types of FMD vaccine seed viruses. However, type Asia1 occurred in North Korea in 2007 and intermittently in neighboring countries [6]. Therefore, the risk of type Asia1 introduction into Korea cannot be excluded. Regarding the type Asia1 outbreak, the government of South Korea has stockpiled the type Asia1 vaccine in the form of an overseas antigen bank. Asia1 Shamir, the most representative Asia1 vaccine, is not highly protective against the Asia1/MOG/05 lineage found in North Korea [7]. Therefore, a novel candidate vaccine, Asia1/MOG/05-R, was previously constructed [8]. 

The capsid of FMDV is composed of four proteins called VP1, VP2, VP3, and VP4. Initially, VP0, a combined form of VP43 and VP2, forms protomers with VP3 and VP1; five protomers come together to form the pentamer of 12S, and 12 pentamers come together to form the viral precursor of 75S. Upon entry of the RNA into the capsid, VP0 is cleaved into VP2 and VP4, creating an intact FMDV (146S) [9]. When subjected to sucrose density gradient ultracentrifugation, 146S is present in the densest position, 75S in a slightly less dense position, and 12S in an even less dense position. FMDV is degraded to 12S by heat or pH, which may not seem like a big difference since the components are the same, but there is a huge difference in immunogenicity between the 146S and 12S forms. According to a previous report [10], the intact FMDV (146S) is more than 100 times more protective against FMDV than the pentameric form (12S). Therefore, in the process of FMD vaccine manufacturing, the yield of FMDV particle production is very important. The antigen productivity of the FMD vaccine plant is directly related to the economics of the plant, leading to the competitiveness of the FMD vaccine.

In this regard, this study aimed to investigate the antigen productivity and virus inactivation kinetics of Asia1/MOG/05-R to validate whether the novel Asia1 vaccine can be economically employed as a candidate vaccine for antigen banks in South Korea.

## 2. Materials and Methods

### 2.1. Cells and Viruses

BHK-21 suspension cells, which were established in serum-free media, as previously described [11], were adjusted to grow in ProVero-1 cell culture medium (Lonza Ltd., Basel, Switzerland) in a shaking incubator at 37 °C with 5% CO_2_ by incubation at 110 rpm. Porcine kidney (LFBK) cells (Plum Island Animal Disease Center, Orient, NY, USA) and BHK-21 adherent cells were cultured in Dulbecco’s modified Eagle’s medium (Thermo Fisher Scientific, Waltham, MA, USA). Recombinant FMDV Asia1/MOG/05-R was constructed by replacing the P1 region of pO-Manisa-FG, which encodes the entire FMDV O1 Manisa/Turkey/69 gene (GenBank accession no. AY593823), with Asia1/MOG/05 (GenBank accession no. EF614458), as previously reported [8]. The virus neutralization tests were performed with Asia1 Shamir (GenBank accession no. JF739177) and Asia1/MOG/2005-R [8].

### 2.2. Determination of Optimal Conditions for Asia1/MOG/2005-R Proliferation

BHK-21 suspension cells were adapted in Cellvento BHK-200 medium (Merck, Darmstadt, Germany) until they reached a density of around 3 × 10^6^ cells/mL for 3.5 days. Then, the Asia1/MOG/05-R was inoculated into the cells at a multiplicity of infection (MOI) of 0.001, 0.005, 0.01, and 0.05 at the time of adding 30% (*v*/*v*) of fresh medium. The cells were incubated in a shaking incubator at 37 °C with 5% CO_2_. Viral infection supernatant was obtained by harvesting at the time of 12, 16, 20, and 24 h post-infection (hpi) and then clarification by centrifugation at 3000× *g* for 20 min at 4 °C to remove cell debris.

### 2.3. Determination of Optimal pH for the Production of the Asia1/MOG/2005-R Antigen

BHK-21 suspension cells (40 mL) were cultured in Cellvento BHK-200 medium (Merck) until they reached approximately 3 × 10^6^ cells/mL for 3.5 days. The pH of the cell culture was adjusted to 6.0, 6.5, 7.0, 7.5, 8.0, 8.5, and 9.0. The Asia1/MOG/05-R was inoculated at 0.05 MOI and incubated in a shaking incubator at 37 °C with 5% CO_2_. The virus supernatant was harvested 24 hpi and then centrifuged at 3000× *g* for 20 min at 4 °C to remove cell debris.

### 2.4. Virus Titration

Adherent BHK-21 cells were used to determine FMDV titers via endpoint titration using the Spearman–Kärber calculation. The results of virus titrations are represented as the tissue culture infectious dose affecting 50% of the cultures (TCID_50_) per mL [12]. 

### 2.5. Quantification of FMDV Particles

The amount of the FMD vaccine antigen (146S) was measured as previously described [13]. Briefly, the viral infection supernatant was treated with chloroform (Merck) at a ratio of 1:1 (*v*/*v*) and mixed vigorously for 5 min. The mixture was then centrifuged at 4000× *g* for 15 min at 4 °C, and then the aqueous phase on top of the organic solvent was collected. Next, the samples were treated with benzonase (Sigma-Aldrich, St. Louis, MO, USA) at a concentration of 0.025 units/μL, and they were incubated at 37 °C for 1 h with shaking. Then, the samples were clarified by centrifugation at 16,000× *g* for 10 min at 4 °C. The FMDV intact particles in the samples were measured by loading the samples onto a high-performance liquid chromatography (Agilent Technologies, Santa Clara, CA, USA) fitted with a TSKgel G4000PWXL column (TOSOH Bioscience, Tokyo, Japan). Finally, the peak area was integrated for the quantification of FMDV particles.

### 2.6. Preparation of Antigen Using A Shaking Flask and 2 L Bioreactor

For flask culture, when the BHK-21 suspension cells with concentrations of 3 × 10^5^ cells/mL at volumes of 28 mL, 140 mL, and 700 mL were grown for 3.5 days up to approximately 3 × 10^6^ cells/mL in a Cellvento BHK-200 medium, the Asia1/MOG/05-R was inoculated onto the cells at 0.05 MOI with 12 mL, 60 mL, and 300 mL of fresh Cellvento BHK-200 medium in a shaking incubator at 37 °C with 5% CO_2_ for 24 h. For the 2 L bioreactor (Sartorius, Göttingen, Germany), 700 mL of the Cellvento BHK-200 cell culture media was initially cultured with a cell density of 3 × 10^5^ cells/mL in a 2 L bioreactor. The bioreactor was fitted with probes to monitor temperature and dissolved oxygen at 37 °C and 45% air saturation. The pH was controlled in the range of 7.2–7.4 by adding CO_2_ and a 0.5 M NaOH solution. The speed of agitation in a bioreactor was maintained at 150 rpm. When the cell density reached around 3 × 10^6^ cells/mL, 300 mL of the cell culture medium was added to the bioreactor, and the pH was adjusted to 7.5. Subsequently, Asia/MOG/05-R was added to the bioreactor containing fresh medium at 0.05 MOI. Viral infection supernatant was harvested after 24 h.

### 2.7. FMDV Inactivation Kinetics

Dissolving bromoethylamine hydrobromide (Sigma-Aldrich) in 10 mL of 0.2 N sodium hydroxide solution (Sigma-Aldrich) resulted in binary ethylenimine (BEI) with a concentration of 0.1 M. Then, the solution was incubated in a shaking incubator at 100 rpm at 37 °C for 1 h. The final pH was adjusted to approximately 8.5–9. The solution was prepared just before experiment. For inactivation kinetics of the samples, 100 mL of the viral infection supernatant was inactivated by adding BEI from 0.5 mM to 3 mM and then was incubated in an incubator at 26 °C and 37 °C at the shaking speed of 75 rpm for 24 h. Next, each sample (12 mL) was collected at the interval of 1 h up to 6 h and 24 h post BEI treatment. Finally, 10% volume of 1 M sodium thiosulfate (Daejung Chemicals, Siheung-si, Korea) to a final concentration of 2% (*v*/*v*) was added to neutralize the residual BEI after experiment. 

### 2.8. Animal Experiment

The purified FMDV Asia1/MOG/05-R antigen (15 μg per dose) derived from the 2 L bioreactor was mixed with 1% saponin (Sigma-Aldrich) and 10% aluminum hydroxide gel (General Chemical, Moorestown, NJ, USA) to prepare a monovalent vaccine. Next, the ISA 206 VG adjuvant (Seppic, Paris, France), pre-warmed at 30 °C, was added at a ratio of 1:1, resulting in a 2 mL/dose of experimental vaccine. The mixtures were incubated at 20 °C for 1 h in a water bath without light exposure and stored at 4 °C until use. Two-month-old pigs (*n* = 5) that had not been previously vaccinated against FMD were immunized twice at the four-week interval with the monovalent Asia1/MOG/05-R vaccine. The control group comprised three unvaccinated pigs. Blood samples were collected at 0, 14, 21, 28, 35, 42, 49, and 56 days post-vaccination. Animal experiments in this study were approved by the Institutional Animal Care and Use Committee (IACUC) of the Animal and Plant Quarantine Agency (IACUC No. 2023-761).

### 2.9. Virus Neutralization Test

The virus neutralization (VN) test was performed as described in the WOAH terrestrial manual [14]. Sera were inactivated at 56 °C for 30 min prior to testing. Starting from 1/4 dilution, 50 μL of two-fold serially diluted sera were mixed with 50 μL of each virus containing 100 TCID_50_. After incubation at 37 °C for 1 h, 50 μL of LFBK cells (0.5 × 10^6^ cells/mL) were added to each well. The plates were sealed and incubated at 37 °C with 5% CO_2_ for 2–3 days. The VN titer was calculated as the reciprocal of the maximum dilution of serum that neutralized the 100 TCID_50_ of the FMDV and expressed as a log_10_ value.

### 2.10. Statistical Analysis

Each experiment was conducted in triplicate, and the means and standard deviations of all values are presented. Statistical data were analyzed using GraphPad Prism version 9 (GraphPad Software, La Jolla, CA, USA) for visual representation. Statistical significance was assessed using two-way ANOVA and set at *p* < 0.05.

## 3. Results

### 3.1. Optimization of Conditions for Asia1/MOG/05-R Proliferation

**Figure 1:**
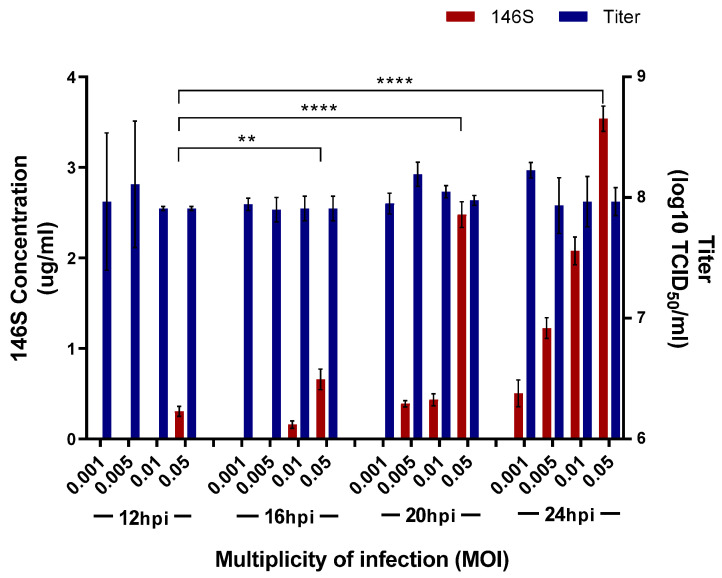
Optimization of the conditions for producing the Asia1/MOG/05-R antigen. The BHK-21 cells were inoculated with Aisa1/MOG/05-R at different virus infection doses and cultivation times, and then antigen yield and titers were measured. Data are presented as the mean ± standard deviation from three independent experiments. hpi: hours post-infection, ** *p* < 0.01, and **** *p* < 0.001.

The FMDV titer and antigen yield were measured according to the Asia1/MOG/05-R concentration and virus infection time to determine the optimal conditions for antigen production using Asia1/MOG/05-R. The viral titer remained constant at approximately 10^8^ TCID_50_/mL under all conditions (Figure 1). However, the amount of antigen increased with a longer virus infection time, peaking at 24 hpi. In addition, the amount of antigen increased with increasing viral concentration. Finally, the optimal condition for antigen production was achieved with a virus concentration of 0.05 MOI and 24 hpi, resulting in an antigen yield of 3.7 μg/mL.

### 3.2. Antigen Yield According to pH Adjustment of the Asia1/MOG/05-R

**Figure 2:**
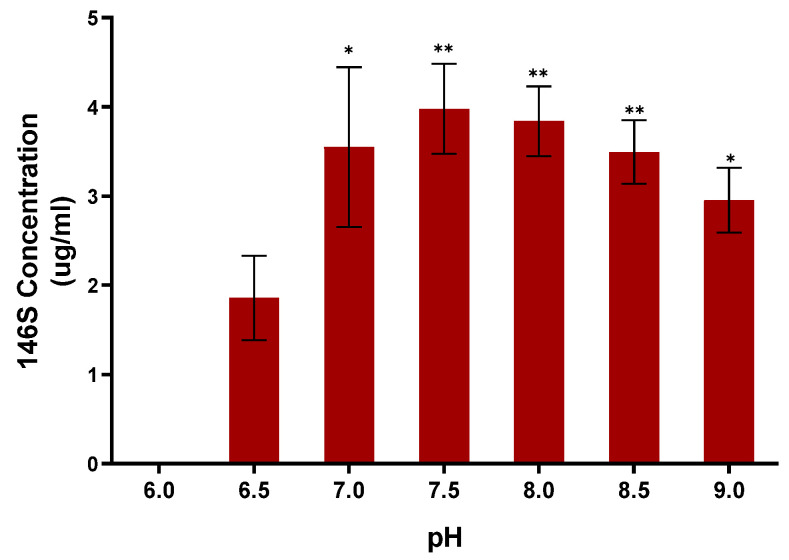
Determination of optimal pH for producing the Asia1/MOG/05-R antigen. The antigen yield was measured according to the medium pH at the virus inoculation stage. Data are presented as the mean ± standard deviation from three independent experiments. * *p* < 0.05, ** *p* < 0.01.

The difference in antigen yield was investigated by changing the medium pH from 6.0 to 9.0 during the Asia1/MOG/05-R inoculation phase. The results showed 0 μg/mL, 1.86 μg/mL, 3.55 μg/mL, 3.98 μg/mL, 3.84 μg/mL, 3.50 μg/mL, and 2.95 μg/mL at pH 6.0, 6.5, 7.0, 7.5, 8.0, 8.5, and 9.0, respectively (Figure 2). The amount of antigen was lowest at pH 6.5 and highest at pH 7.5, followed by a decreasing trend with increasing pH. Finally, the optimal condition for Asia1/MOG/05-R antigen production was a medium pH of 7.5 at the time of virus inoculation.

### 3.3. Comparison of Antigen Yield According to Production Scale

**Figure 3:**
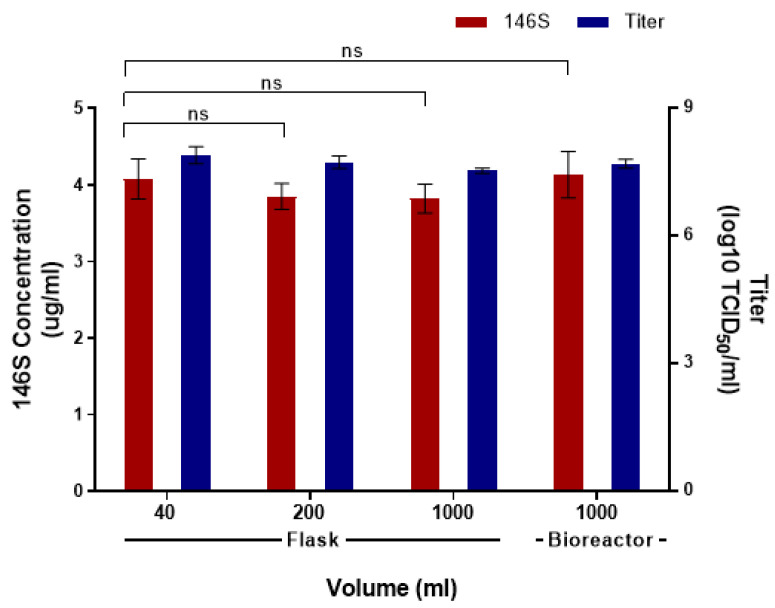
Comparison of antigen yield according to the production scale. Asia1/MOG/05-R was produced by progressively increasing the culture volume 5-fold, and antigen yield and titer were measured at each step. Data are presented as the mean ± standard deviation from three independent experiments. ns: not significant.

The amount of antigen for the Asia1/MOG/05-R was evaluated by culturing in the volumes of 40 mL, 200 mL, and 1000 mL, resulting in 4.1 μg/mL, 3.85 μg/mL, and 3.82 μg/mL, respectively. Viral titers were >10^7^ TCID_50_/mL, regardless of the volume of viral proliferation (Figure 3). When the Asia1/MOG/05-R antigen was produced in a bioreactor based on the optimal conditions established at the flask scale, the virus titer exceeded 10^7^ TCID_50_/mL, and the antigen yield was 4.1 μg/mL.

### 3.4. BEI Inactivation Kinetics of the Asia1/MOG/05-R

**Figure 4:**
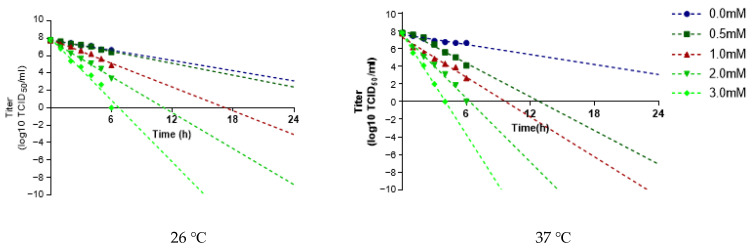
Inactivation kinetics of Asia1/MOG/05-R. The supernatant obtained after the Asia1/MOG/05-R was inoculated in the BHK-21 suspension cells was inactivated by each binary ethyleneimine (BEI) concentration, with samples taken at intervals of 1 h up to 6 h and 24 h. The individual graph was extrapolated with a linear line for the analysis of inactivation kinetics.

The inactivation kinetics of the virus were investigated using the Asia1/MOG/05-R infection supernatant obtained from the flask. The inactivation rate was greater at 37 °C than at 26 °C, and the higher the BEI concentration, the greater the inactivation rate. While the viral titer was estimated to be <10^−7^ TCID_50_/mL with linear regression within 24 h with 2 mM BEI at 26 °C, inactivation was completed by reducing the viral titer to 10^−7^ TCID_50_/mL within 24 h with 0.5 mM BEI at 37 °C (Figure 4). The amount of antigen was measured immediately before and 6 h and 24 h after BEI inactivation. Treatment with 3 mM BEI showed antigen yields of 5.0 and 5.1 µg/mL at 26 °C and 37 °C, respectively, from the initial antigen yield of 5.7 µg/mL. However, this loss was not due to BEI treatment because the samples without BEI treatment also showed equivalent amounts of antigen (5.2 µg/mL) under the same conditions (Table 1).

### 3.5. Immunogenicity of the Asia1/MOG/05-R in Pigs

**Figure 5:**
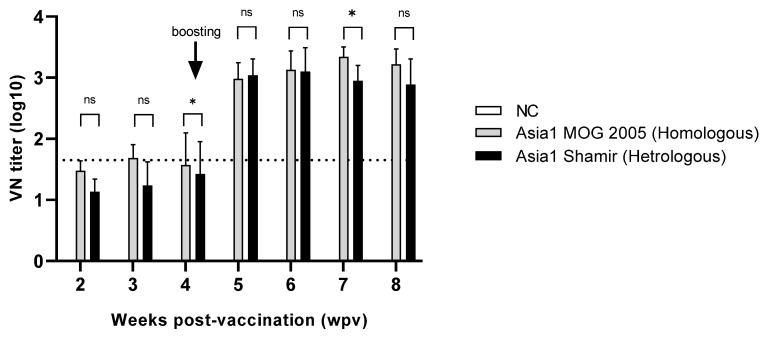
Virus neutralization antibody titers post-immunization with the Asia1/MOG/05-R vaccine. Virus neutralization tests against the Asia1/MOG/05-R and Asia1 Shamir viruses were performed using sera collected weekly from pigs immunized twice with the Asia1/MOG/05-R vaccine at the four-week interval. Data are presented as the mean ± standard deviation from three independent experiments. The dotted line indicates 1.65 log10 (1:45) virus neutralization (VN) titer. * *p* < 0.05, ns: not significant.

Pigs were inoculated with a 15 µg/dose of the Asia1/MOG/05-R antigens produced from the bioreactor. VN titers against Asia1/MOG/05-R and Asia1 Shamir viruses were approximately 1:45 or lower after the first vaccination. However, after the second immunization, the VN titers increased and reached approximately 1:1000 (Figure 5). There was no significant difference in the VN titers between the Asia1/MOG/05-R and Asia1 Shamir viruses.

## 4. Discussion

A recombinant Asia1/MOG/05 vaccine (Asia1/MOG/05-R) was developed at our institute [8]. To evaluate the feasibility of using Asia1/MOG/05-R in antigen banks, its antigen productivity and virus inactivation kinetics were investigated. In addition, the immunogenicity of the antigen produced in the bioreactor was evaluated in pigs.

The optimal conditions for Asia1/MOG/05-R antigen production were determined using the Cellvento BHK-200 medium, which does not require medium exchange during the virus inoculation stage. FMDV titers were equivalent at approximately 10^8^ TCID_50_/mL under all conditions. However, the antigen yield was higher at higher virus concentrations and longer virus infection times. There was a difference in antigen yield even at similar titers; however, the reason for this is unclear. Meanwhile, the efficacy of the FMD vaccine depends on the 146S intact virus particle content rather than the virus titer; therefore, antigen content is important. Other researchers have reported that high viral titers but low antigen amounts lead to low immunogenicity in animals [15].

The antigen yield of Asia1/MOG/05-R varied depending on the pH of the virus inoculation medium and was highest at pH 7.5. To date, there have been no reports comparing antigen productivity based on pH variations in this way. However, a previous study analyzed the physical stability of FMDV according to pH and reported that FMDV was most stable at pH 8 [16,17]. Therefore, although there is a correlation between the stability and antigen productivity of FMDV, it is not completely consistent. 

When the Asia1/MOG/05-R was scaled up in 5-fold increments in the flask level, antigen yield was approximately 4 μg/mL regardless of antigen production scale. This was a markedly higher amount than the antigen amount of 1 to 3 μg/mL reported in previous studies [18,19,20,21]. Notably, when Asia1/MOG/05-R was grown in the bioreactor, the same amount of antigen was produced in the flask. Virus growth in flasks is higher than that in bioreactors [22], whereas similar antigen yields have been reported at both scales [23]. Although this is the result of cultivating the virus at half capacity (1 L) of a 2 L bioreactor, the antigen amount of 4 μg/mL is more productive than other foreign cases. FMD vaccine manufacturing facilities use industrial-scale bioreactors larger than 1000 L; however, when applying other viruses, the amount of antigen production is similar even when smaller bioreactors are used [24,25]. This indicates that Asia1/MOG/05-R is a commercially valuable vaccine strain because it can lower the cost of vaccine production. 

Because FMDV is produced in large quantities in a specially sealed facility called biosafety level 3, it is necessary to investigate the innocuity of the final antigen and the inactivation kinetics during antigen production [26]. During inactivation of the virus, times samples should be taken at regular intervals for the purpose of monitoring the rate and linearity of the inactivation process. The log10 infectivity of the timed samples are plotted against time, and the inactivation procedure is not considered satisfactory unless at least the latter part of the slope of the line is linear and extrapolation indicates that there would be less than one infectious particle per 104 liters of liquid preparation at the end of the inactivation period [14]. FMD vaccine antigens, especially, could be degraded by enzymes derived from the cell when they were exposed to 37 °C for a long period [27]. According to the 2009 edition of the OIE manual, the inactivation kinetics of FMDV could be carried out at 26 °C or 37 °C. On the basis of the previous report, we performed the inactivation kinetics of Asia1/MOG/05-R at both temperatures depending on BEI concentrations. Consistent with the results of the present study, several studies have reported that higher temperatures lead to better viral inactivation [28,29]. Inactivation was complete with a drop in viral titer to 10^−7^ TCID_50_/ml within 24 h with 2 mM BEI 37 °C, consistent with previous results [28]. At 37 °C, inactivation was complete with 0.5 mM BEI, consistent with previous reports of inactivation at 0.5 mM or 1.0 mM BEI for other viruses [27]. In the samples immediately prior to inactivation and at 6 h and 24 h after 3 mM BEI treatment, approximately 10% of the antigen was lost regardless of the BEI treatment. This antigen loss during storage was relatively low compared to other previous reports that 1 mM BEI treatment at 37 °C resulted in losses of >60% of initial antigen [29]. In certain cases, antigen loss reached 80% during 24 h of storage at 37 °C even without BEI treatment [30]. This indicates that Asia1/MOG/05-R is relatively more stable than other foreign FMD vaccine strains. 

Two immunizations of pigs with the Asia1/MOG/05-R antigen prepared in a bioreactor resulted in a VN titer of approximately 1:1000, which is predicted to be protective against the two Asia1 lineages based on data reporting a correlation between antibody levels and animal protection [31]. 

Bovine sera obtained after immunization with the Asia1 Shamir vaccine showed low vaccine-matching values for the Asia1/MOG/05 virus [32]. In this study, VN titers against the Asia1/MOG/05-R and Asia1 Shamir viruses were not significantly different, which is in line with previous results showing that swine sera from one and two immunizations with a commercial Asia1 Shamir vaccine had equivalent VN titers against the Asia1/MOG/05 and Asia1 Shamir viruses [7]. Since a good vaccine candidate should ensure a high and long-lasting immune response, further investigation to evaluate immunity duration needs to be explored in the future.

In conclusion, Asia1/MOG/05-R may be a useful vaccine strain for antigen banks when FMD vaccine manufacturing facilities are established in South Korea.

## Figures and Tables

**Table 1:** The amount of foot-and-mouth disease vaccine antigen (146S) in the Asia1/MOG/05-R virus supernatants after binary ethylenimine (BEI) treatment at 26 °C and 37 °C for 6 h and 24 h.

BEI Concentration	Antigen Amount (μg/mL) with Different Temperatures and Times					
	26 °C			37 °C		
	0 h	6 h	24 h	0 h	6 h	24 h
**0.0 mM**	5.7 ± 0.25	5.8 ± 0.10	5.2 ± 0.05	5.7 ± 0.25	5.8 ± 0.07	5.2 ± 0.10
**0.5 mM**	5.7 ± 0.25	5.7 ± 0.08	5.2 ± 0.18	5.7 ± 0.25	5.6 ± 0.20	5.2 ± 0.21
**1.0 mM**	5.7 ± 0.25	5.6 ± 0.28	5.2 ± 0.15	5.7 ± 0.25	5.6 ± 0.22	5.1 ± 0.11
**2.0 mM**	5.7 ± 0.25	5.6 ± 0.10	5.1 ± 0.11	5.7 ± 0.25	5.8 ± 0.26	5.2 ± 0.31
**3.0 mM**	5.7 ± 0.25	5.7 ± 0.12	5.0 ± 0.15	5.7 ± 0.25	5.5 ± 0.28	5.1 ± 0.17

## Data Availability

Data are retained within this article; The raw data are available from the corresponding author.
